# Guidelines and treatment for illicit drug related presentations in emergency departments: A scoping review

**DOI:** 10.1177/10398562231191671

**Published:** 2023-08-07

**Authors:** Ruby Tszwai Au, Elizabeth Hotham, Vijayaprakash Suppiah

**Affiliations:** UniSA Clinical and Health Sciences, 1067 University of South Australia, Adelaide, SA, Australia; UniSA Clinical and Health Sciences, 1067 University of South Australia, Adelaide, SA, Australia; UniSA Clinical and Health Sciences, 1067 University of South Australia, Adelaide, SA, Australia; Australian Centre for Precision Health, 1067 University of South Australia, Adelaide, SA, Australia

**Keywords:** illicit drug-induced psychosis, emergency department, psychotic disorders, clinical guidelines, pharmacological treatment

## Abstract

**Objective:**

This review aimed to identify current pharmacological and non-pharmacological treatment employed in emergency departments (EDs) for the management of patients presenting with illicit drug-related presentations (IDP) and compare current treatments with recommendations provided in guidelines.

**Method:**

The review consists of English peer-reviewed journal articles and grey literature published in electronic databases: Ovid MEDLINE, PubMed, Embase Classic+Embase, Ovid Emcare and APA PsycInfo between 2015 and 2022.

**Results:**

Twelve studies were identified from the search, with agitation and aggression being the most common presentations, and cannabis being the most prevalent illicit drug. Ventilatory support and restraints were the most reported non-pharmacological interventions while benzodiazepines and antipsychotics were the most commonly prescribed pharmacological agents. Non-coercive de-escalation strategies were recommended in all guidelines, with verbal de-escalation being the initial approach before other interventions, such as medications and restraints. However, de-escalation strategies were not reported in any studies.

**Conclusions:**

Pharmacological interventions for patients with IDP and related symptoms were in accordance with guidelines. Use of restraints was identified in included studies with notable lack of reporting of de-escalation strategies which may have been deemed insignificant and not reported. Future research could investigate the appropriateness of restrictive interventions as well as the employment of non-restrictive de-escalation strategies.

‘Illicit drug use’ encompasses the use of prohibited psychoactive substances (primarily cannabis, amphetamine, ecstasy and heroin), psychoactive pharmaceuticals for non-medical purposes and inappropriate use of substances, such as glue, petrol and paints.^1,2^ A recent Australian survey reported that nine million Australians >14 years had used illicit drugs at least once in their lifetime.^3^ Other surveys have indicated that cannabis, cocaine, methamphetamine, ecstasy and heroin were easily obtainable.^4,5^

Cannabis, cocaine and crystal methamphetamine (CMA) have been associated with increased risk of acute psychosis and exacerbation of psychotic symptoms.^7–10^ Recent users (31%) of CMA developed mental health issues, similar to users of cannabis (27%), ecstasy (22%) and cocaine (22%) respectively.^3^ This risk was three-fold higher in recent users of CMA when compared to the general population.^9^

Illicit drug-induced psychosis (IDP) poses a significant burden on emergency departments (EDs) and health systems.^11,12^ Individuals with IDP are often agitated, and experience hallucinations and delusions,^13^ requiring additional care and behaviour management. A longitudinal study over 4 years showed that the most common clinical presentations among ED patients with acute drug toxicity are agitation and aggression.^14^ Of these, 71.3% of the presentations were transported by ambulances with 88.9% discharged within 24 h, and approximately 45% not needing treatment over and above observation.^14^ A Western Australian Parliamentary report calculated that approximately a third of 29 ED cubicles were occupied for 11 h daily by IDP-affected individuals presenting with general agitation, distress and violent and aggressive behaviour.^15^

Despite the established relationship between illicit drug use and acute psychosis, less is known on treatments provided to ED patients with IDP. This review aims to (i) examine current pharmacological and non-pharmacological treatments recommended in guidelines for management of IDP patients and (ii) compare treatments and interventions in EDs with guidelines.

## Methods

Four key concepts were identified: illicit drugs, IDP, EDs, treatments and interventions. Electronic databases: Ovid MEDLINE, PubMed, Embase Classic + Embase, Ovid Emcare and APA PsycInfo were searched with a combination of medical subject headings (MeSH) and specific keywords (non-MeSH terms).

Search terms used for the first key concept were: illicit drugs, including: illicit drug, illegal drug, recreational drug, amphetamine, cocaine, cannabis and for the second key concept, IDP: psychotic disorder, psychosis, psychotic, psychoses. Search terms for ED presentations were: emergency service, emergency department, emergency ward, emergency room and treatment and intervention used for the last key concept. Search terms for each key concept were joined using the Boolean operator OR and combined with AND, creating a search strategy to narrow the search results. Google Scholar was searched for reports and documents from government and non-government organisations. Further relevant citations were identified by searching reference lists of identified papers.

## Results

Journal articles in English published between 2015 and 2022 were included, with 332 manuscripts identified from the five databases and 34 additional papers from Google Scholar. A total of 59 duplicates and 38 reviews and papers where full text could not be accessed were removed. After the addition of five papers from reference lists, a total of 75 manuscripts were reviewed and independently verified. Sixty-three papers unrelated to IDP and related symptoms and without reported treatments and interventions in EDs were excluded.

The most recent guidelines from Australia,^16–18^ the UK^19,20^ and the US^21–26^ (Table 1) and eleven original studies^27–37^ and a transcript of evidence^15^ (Table 2) were included in this review.

**Table 1.** Guidelines for the management of acute behavioural disturbances and acute psychosis

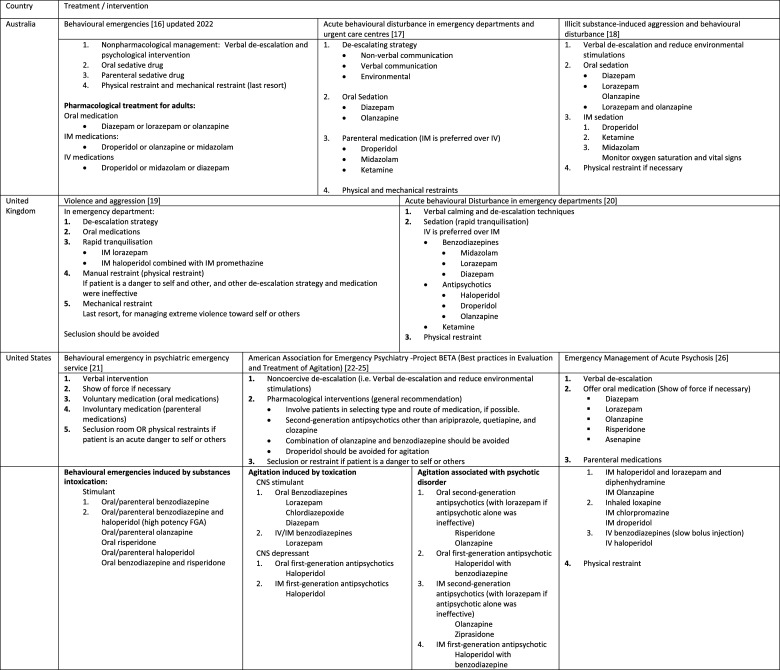

IM: Intramuscular; IV: intravenous.

**Table 2.** Treatments and interventions for individuals with illicit drug-induced psychosis

Country	Study period	Population (n)	Setting	Substances used	Clinical presentation (%)	Treatment/intervention (%)	Reference
Observational studies							
Australia	1 January 2015–31 December 2015	Patients with ATS-related presentations (*n* = 189)	ED Mental Health Team	ATS With cannabis, alcohol, tobacco, heroin, BZDs, cocaine, LSD, GHB, prescription opioid or other prescription medications	Psychosis (34.4) Suicidal (27) Aggression (12.2) Anxious (3.2) Depression (2.6) Self-inflicted lacerations (2.1)	Oral BZDs (15.9) Parenteral BZDs (13.8) Oral antipsychotics (11.1) Parenteral antipsychotics (14.8)	^27^
Australia	July 2005–June 2016	First admission of patients with amphetamine induced psychosis (*n* = 6130) (control: *n* = 41,444)	ED presentations, hospital admissions and community mental health contact	Amphetamine	Presentation not reported	Early intervention • Routine assessment • Employment of consultation liaison psychiatrists • CAARMS	^28^
Australia	September 2017	Patients with methamphetamine-related presentations (*n* = 95)	ED and AIS	Methamphetamine	ED Aggression towards staff (65.4), toward other patients (50) AIS Aggression toward staff (53.3), toward other patients (20)	ED • Physical restraint (69.2) • mechanical restraint (61.5) AIS • Physical restraint (13.3) • mechanical restraint (6.7) Seclusion room (13.3)	^29^
Poland	1 January 2010–31 December 2016	Patients with psychoactive substance-induced psychotic disorder (*n* = 156)	EDs and Psychiatric wards	Amphetamine Opioids Ecstasy Cannabis/synthetic cannabinoids	Delusional (63.5) Hallucinations (50.0) Aggressive behaviour (60.3) Anxiety and agitation (33.3) Mood disturbances (60.9) Suicidal thoughts (14.1) Self-mutilation (3.9) loquacity (21.8) Disassociation (19.2) Silliness (4.5) Behaving oddly (3.2) Psychomotor related (56.4)	BZDs (34.3) Antipsychotics (8.6) Mood stabilisers (12.5) Antidepressant (1.9)	^30^
United States	1 January 2010–30 June 2013	Adolescents with substances related presentations (*n* = 202 (illicit drug = 101))	ED	Illicit drugs: Cannabis LSD Cocaine Ecstasy Heroin Synthetic cannabinoids Synthetic cathinones Other: Prescription stimulants (amphetamine), anticholinergics, opioids, BZDs, ethanol, analgesic, antidepressants, antipsychotics	Central nervous system manifestations (80.4) Acute psychosis or delirium (38) Agitation (30) CNS depression (25.8)	(Illicit drug only) Routine supportive care (100) Neurologic management (BZDs (34.3), antipsychotics (8.6), anticonvulsants (3.8), vasopressors (1.0), bronchodilator (1.0), sodium bicarbonate (1.0) Non-pharmacological support (Intravenous fluid resuscitation (18) and Intubation/ventilatory support (9.5)) Antidotes (Naloxone/nalmefene (5.7), N-acetylcysteine (1.9), Physostigmine (3.8)) Gastrointestinal decontamination (bowel irritation (1.9), active charcoal (1.9)) Bedside consultation (∼7)	^31^
Case reports							
Australia	Study period not recorded	Patient presenting with severe acute psychotic relapse due to substance use (*n* = 1)	ED and psychiatric ward	Cannabis Methamphetamine	Aggressive and combative behaviour	Six-point mechanical restraint 1 L of 0.9% normal saline solution Sedative (ziprasidone, lorazepam, droperidol, zuclopenthixol acetate and chlorpromazine syrup) Benztropine Seclusion room Lorazepam and droperidol administrated under physical restraint	^32^
Spain	June 2014–May 2015	Patients with substance-induced psychotic disorder (*n* = 2)	ED/hospital	Case 10 Alcohol, nicotine and cannabis Case 11 Alcohol, nicotine, cocaine, cannabis and amphetamine	Acute psychotic agitation	Inhaled loxapine	^33^
Europe	1 October 2013–31 September 2014	Patients with GHB or GBl related presentations (*n* = 710)	ED and hospital	GHB or GBL With ethanol, amphetamine, cocaine, BZDs, cannabis, heroin, ketamine, methadone or LSD	Anxiety (14.8) Agitation and aggression (37.9) Hallucination (4.6) Behaviour abnormality (include psychosis) (39.6) Severe depression of consciousness (35.4)	Received no treatment (43.4) Supportive treatment only- intravenous fluid therapy (31.5) Sedative (19.9) • BZDs (15.4) Antidote treatment (21) • Naloxone (20.3) • Flumazenil (5.4) Endotracheal intubation and mechanical ventilation (6.9)	^34^
Europe	1 October 2013–31 March 2014	Patients with cannabis-related presentations (*n* = 35)	ED	Cannabis alone	Agitation and aggression (22.9) Psychosis (20) Anxiety (20) Hallucinations (8.57)	Received no treatments (71.4) Sedative (17.14) • BZDs (11.4) • Olanzapine (5.7) • Chlorprothixene (2.8) • Hydroxyzine (8.6) 20% psychotic patients Received no sedation/other pharmacotherapy (71) Olanzapine (29)	^35^
Switzerland	1 October 2014–30 September 2015	Patients with psychoactive substance-related presentations (*n* = 210)	ED	Cannabis Cocaine Opioid BZDs Meth/amphetamine Ecstasy Methadone Heroin Ketamine LSD GHB Other prescription medicines	Anxiety, nervousness or fear (23) Agitation or aggression (17) Hallucination (4) Psychosis (3) Suicide ideation (<1)	Intravenous fluid administration (72) Tracheal intubation (3) Sedative (BZDs, antipsychotics, propofol and ketamine) (24) Antidote (naloxone, flumazenil and biperiden) (9)	^36^
United States	Study period not recorded	Patient with cannabis-induced psychotic disorder and severe marijuana use disorder (*n* = 1)	ED and PES	Cannabis	Acute psychosis Erratic and disruptive behaviour Auditory hallucinations Paranoia	Two-point soft restraints Physical restraints Risperidone Lorazepam	^37^
Transcript of evidence							
Australia	Year not reported	Patients with IDP (number not reported)	EDs	Cannabis Synthetic cannabinoids Meth/amphetamine Heroin Ecstasy Synthetic cathinone	Agitation and aggression Distress Psychosis Trauma Violence	Sedating medications Physical restraint Ventilation Naloxone Verbal de-escalation	^15^

AIS: Acute Psychiatric Inpatient Service; ATS: Amphetamine type stimulants; CAARMS: Comprehensive Assessment of At-Risk Mental States; CBT: Cognitive behavioural therapy; CNS: Central nervous system; CRT: Cognitive remediation therapy; ECT: Electroconvulsive therapy; ED: Emergency department; GBL: Gamma-butyrolactone; GHB: Gamma-hydroxybutyrate; IDP: illicit drug-induced psychosis; LSD: Lysergic acid diethylamide; PES: psychiatric emergency service.

### Current guidelines

ED presentations associated with substance use often involved agitated and aggressive patients requiring rapid tranquilisation and urgent treatment. However, guidelines that included recommendations for managing IDP generally focused on schizophrenia, full-blown psychosis and early interventions, rather than agitation and aggression, with little being ED-specific.

Most guidelines (Table 1) focussed on managing acute behavioural disturbance, violence and aggression in EDs, with limited emphasis on specific recommendations for substance-induced presentations.

Even though there were variations in pharmacological interventions between guidelines, non-pharmacological strategies were almost identical: non-restrictive strategies first line with physical restraints employed as last resort.

All guidelines included benzodiazepines (BZDs) such as diazepam and lorazepam and antipsychotics such as droperidol and olanzapine, with midazolam and ketamine in the Australian^17,18^ and UK^20^ guidelines only, and haloperidol in all except the Australian guidelines.^19–26^

Risperidone was only recommended in the US guidelines.^21–26^ Droperidol was recommended in most guidelines for managing acutely agitated individuals,^16–18,20,26^ except for the American Association for Emergency Psychiatry.^22–25^ The NICE guidelines included antihistamines and suggested combining promethazine and haloperidol for rapid tranquilisation.^19^ The US guidelines recommended multiple medications not reported in other guidelines.^21–26^

Although specific medications differed between guidelines, they were generally antipsychotics, benzodiazepines or antihistamines, with varying routes of administration. The Australian Therapeutics Guidelines suggested that in EDs, intravenous (IV) route was preferable to intramuscular (IM) and the latter used if patients failed to cooperate.^16^ In comparison, both the Victorian and New South Wales guidelines^17,18^ recommended oral medications as first line, followed by IM. The NICE guidelines^19^ suggested IM medications for rapid tranquilisation, while The Royal College of Emergency Medicine Best Practice Guideline^20^ recommended IV for quicker onset of action.

The US expert consensus guideline recommended both oral and parenteral medications for behavioural emergency related to stimulant intoxication, without specifying the parenteral route.^21^ The project BETA guideline^24^ recommended oral and IM medications and if possible, involving patients in deciding the types and routes of medication. Lastly, Freudenreich (2020) recommended the oral route over IM, and IV as third line.^26^

### Clinical presentations

Of the symptoms charted on clinical presentations, agitation and aggression^15,27,29–36^ and psychosis^15,27,31,33–37^ were most frequently reported, followed by hallucination,^30,34–37^ anxiety,^27,30,34–36^ suicidal ideation^27,30,36^ and delusion.^30^ Other clinical presentations reported included: cardiovascular, respiratory and renal system manifestations, and gastrointestinal and hepatic symptoms.^31,34–36^

### Commonly reported substances

The most prevalent illicit drug reported was cannabis,^15,27,30–37^ followed by CMA and other amphetamines,^15,28–34,36^ cocaine,^27,31,34,36^ ecstasy,^15,30,31,36^ heroin^15,27,31,34,36^ and lysergic acid diethylamide (LSD).^27,31,34,36^ Others included gamma-hydroxybutyrate (GHB) and gamma-butyrolactone (GLB),^27,34,36^ synthetic cannabinoids and cathinones.^15,30,31^ Concurrent use of multiple psychoactive substances was common,^27,30–34,36^ with illicit drugs used in combinations with legal substances such as alcohol, nicotine and caffeine,^33^ and prescription medications, including BZDs, opioids, ketamine, antidepressants, anticholinergics and antipsychotics.^15,27,30,31,33,34,36^ Patients presenting at EDs were often unable to identify substances used, or identified them incorrectly, heightening risk of adverse effects due to drug interactions.^30^

### Treatment for IDP

#### Non-pharmacological interventions

Apart from physical restraints, ventilatory support, intravenous fluid replacement,^31,32,34,36^ seclusion,^29,32^ routine supportive care and bedside consultations^31^ were the most reported non-pharmacological interventions.^15,29,31,32,34,36,37^

#### Pharmacological interventions

BZDs^27,31,32,34–37^ and antipsychotics^27,30–33,35–37^ were the most used pharmacological agents with two case reports specifying lorazepam.^32,37^ Antipsychotics have been prescribed for psychosis management, agitation and aggressive behaviour^27,30,37^ with reports of both typical (first generation) antipsychotics – droperidol, zuclopenthixol, chlorpromazine,^32^ loxapine^33^ and chlorprothixene^35^ – and atypical (second generation) antipsychotics – olanzapine, ziprasidone and risperidone.^32,35,37^

Other treatments included antidepressants,^30^ mood stabilisers,^30^ anticonvulsants,^31^ vasopressors,^31^ bronchodilators,^31^ sodium bicarbonate,^31^ benztropine,^32^ propofol^36^ and antihistamines.^35^

Antidotes, predominantly naloxone and flumazenil, were often prescribed for opioid and BZD overdose, respectively.^15,31,34,36^ Others such as biperiden for drug-induced extrapyramidal symptoms,^36^ nalmefene for opioid overdose, n-acetylcysteine for paracetamol overdose and physostigmine for anticholinergic toxicity were also reported.^31^

However, not all ED presentations involved pharmacological treatment. In one study of cannabis-related presentations, 71.4% of patients received no treatment, given self-resolving symptoms.^35^ Another study also found that over 40% of presentations after GHB or GBL use did not receive pharmacological treatment.^34^

## Discussion

To our knowledge, this is the first review to compare publicly available guidelines for the management of illicit drug-related ED presentations in Australia, the UK and the US with current treatments employed in EDs as reported in pertinent literature.

Among the five Australian papers,^15,27–29,32^ only one focused on identifying opportunities for implementing early interventions to prevent substance-induced psychosis.^28^ Another^27^ reported on use of oral or parenteral BZDs and antipsychotics in line with Australian guidelines (Table 1), without specifying individual medications. The remaining three papers reported using restraints as one of the interventions.

Unadkat et al. (2019) reported use of physical and mechanical restraints in over half of the methamphetamine-related ED presentations.^29^ The appropriateness was difficult to assess as pharmacological interventions and other de-escalating strategies were not reported. Tucker et al. (2016) reported a case of use of ziprasidone, oral lorazepam, droperidol, zuclopenthixol acetate, chlorpromazine and benztropine,^32^ with only droperidol concordant with Australian guidelines.^16–18^ This patient had a complex treatment history and therefore the use of lorazepam and zuclopenthixol may be justified. Benztropine was also administered to minimize extrapyramidal side effects.^32^ The only Australian literature to report use of verbal de-escalation was the transcript of evidence from the Western Australian Parliament.^15^

Even though five European studies^30,33–36^ were included in this review, only UK guidelines could be sourced.^19,20^ Hence, the appropriateness of reported interventions in these studies may not be fully determined. Both UK guidelines recommended non-restrictive de-escalation strategies, use of BZDs (lorazepam, midazolam or diazepam), antipsychotics (haloperidol, droperidol, olanzapine) and ketamine. Medications reported in the European studies were from the same classes as UK guidelines and antidotes prescribed for substance overdose were appropriate for intoxicated individuals.^34,36^ However, the latter were not included in the UK guidelines. Restrictive interventions were not reported in the European studies,^30,33–36^ potentially due to lack of recording.

A US case study^37^ reported use of risperidone, lorazepam, and both physical and mechanical restraints for cannabis-induced acute psychosis. While use of oral risperidone and lorazepam adhered to guidelines,^24,26^ recommended oral medications for agitation induced by CNS depressant intoxication were first-generation antipsychotics, such as haloperidol.^24^ Lack of reporting on de-escalation strategies makes it challenging to comment on the use of restraints. Lastly, Finkelstein et al. (2017) reported use of BZDs, antipsychotics and other medications as well as supportive therapies like antidotes, anticonvulsants and ventilatory support.^31^ However, the latter were not recommended in US guidelines, despite specific sections addressing substance intoxication.^21–25^

All guidelines recommended BZD use (Table 1) although the preferred initial approach for agitation and aggression were verbal and other de-escalation strategies, including offering food and reducing environmental stimulation (Table 1). Restraints were frequently reported as a non-pharmacological intervention,^15,29,32,37^ despite guidelines advising their use as last resort when there is risk of harm to self or others. Despite non-coercive de-escalation strategies being recommended as first line, they were only reported in the transcript of evidence.^15^ It is uncertain whether restraints were considered after other attempts to de-escalate were ineffective or used incorrectly, or not reported as they were not considered as interventions. Hence, the prevalence of verbal de-escalation and its role in the clinical setting remains unclear.

Ventilatory support and antidotes were also commonly reported. Antidotes essential for intoxicated individuals were not recommended in any guidelines. Urine drug screens may lack accuracy and speed for rapid decision making in EDs,^38,39^ but could be useful for those acutely psychotic, or unable to provide a reliable history.^40^

### Limitations

While most studies aligned with recommended medication classes in guidelines, medication dosages were rarely reported, making direct comparisons challenging. Although pharmacological interventions were concordant with guidelines, the use of restraints remained unclear as verbal de-escalation strategies were not reported. Restrictive interventions were not reported in the European studies, suggesting limited usage, but this assumption may not be reliable due to the small number of studies and the uneven distribution between countries. The lack of detailed information on treatments, reasons for employing restrictive interventions, early use of non-coercive strategies and management strategies for different illicit drugs are other limitations, noting that CMA-induced presentations tend to more challenging and require immediate attention compared to other substances.^41^ Future research could compare outcomes for patient recovery and staff/environmental safety based on different approaches and the differences in managing IDP in patients with underlying psychiatric disorders compared to those without.

## Conclusion

Cannabis was the most frequently reported illicit drug while aggression and agitation were the most reported clinical presentations. Despite guidelines recommending non-coercive de-escalation strategies as first line, none of the studies reported their use. Restraints and ventilatory support, and benzodiazepines and antipsychotics were the most reported non-pharmacological and pharmacological interventions, respectively. The use of physical restraints in Australian and US studies highlighted the lack of documentation of prior de-escalation strategies. Future research could investigate the rationale behind using restrictive interventions as well as the use of verbal and other de-escalation strategies in EDs.
